# Primary Care Doctor Characteristics That Determine the Use of Teleconsultations in the Catalan Public Health System: Retrospective Descriptive Cross-Sectional Study

**DOI:** 10.2196/16484

**Published:** 2020-01-31

**Authors:** Oscar Solans Fernández, Francesc López Seguí, Josep Vidal-Alaball, Josep Maria Bonet Simo, Oscar Hernandez Vian, Pascual Roig Cabo, Marta Carrasco Hernandez, Carmen Olmos Dominguez, Xavier Alzaga Reig, Yesika Díaz Rodríguez, Manuel Medina Peralta, Eduardo Hermosilla, Nuria Martínez León, Nuria Guimferrer, Mercedes Abizanda González, Francesc García Cuyàs, Pol Pérez Sust

**Affiliations:** 1 Health Department Catalan Ministry of Health Barcelona, Catalonia Spain; 2 TIC Salut Social Ministry of Health Barcelona, Catalonia Spain; 3 Center for Research in Health and Economics Pompeu Fabra University Barcelona, Catalonia Spain; 4 Health Promotion in Rural Areas Research Group Gerència Territorial de la Catalunya Central Institut Català de la Salut Sant Fruitós de Bages, Catalonia Spain; 5 Unitat de Suport a la Recerca de la Catalunya Central Fundació Institut Universitari per a la recerca a l'Atenció Primària de Salut Jordi Gol i Gurina Sant Fruitós de Bages, Catalonia Spain; 6 Fundació Institut Universitari per a la recerca a l'Atenció Primària de Salut Jordi Gol i Gurina Barcelona, Catalonia Spain; 7 Parc Sanitari Pere Virgili Ministry of Health Barcelona, Catalonia Spain; 8 Sant Joan de Déu Hospital Catalan Ministry of Health Barcelona, Catalonia Spain

**Keywords:** tele-medicine, tele-consultation, remote consultation, primary care, general practitioners

## Abstract

**Background:**

eConsulta is a tele-consultation service involving doctors and patients, and is part of Catalonia's public health information technology system. The service has been in operation since the end of 2015 as an adjunct to face-to-face consultations. A key factor in understanding the barriers and facilitators to the acceptance of the tool is understanding the sociodemographic characteristics of general practitioners who determine its use.

**Objective:**

This study aimed to analyze the sociodemographic factors that affect the likelihood of doctors using eConsulta.

**Methods:**

A retrospective cross-sectional analysis of administrative data was used to perform a multivariate logistic regression analysis on the use of eConsulta in relation to sociodemographic variables.

**Results:**

The model shows that the doctors who use eConsulta are 45-54 years of age, score higher than the 80th percentile on the quality of care index, have a high degree of accessibility, are involved in teaching, and work on a health team in a high socioeconomic urban setting.

**Conclusions:**

The results suggest that certain sociodemographic characteristics associated with general practitioners determine whether they use eConsulta. These results must be taken into account if its deployment is to be encouraged in the context of a public health system.

## Introduction

The use of tele-consulting, synchronous or asynchronous consultation using information and communication technologies (ICT) to omit geographical and functional distance between general practitioners and citizens in primary health care, is widespread in both public [1,2] and private [3] medicine. Although various studies suggest it is beneficial in certain contexts such as the monitoring of diabetes, heart disease, and high blood pressure [4,5] and well accepted by patients [6], its uptake remains low [7], and there are difficulties facing its use in clinical practice [8,9]. Some studies have pointed out that these difficulties may be due to a lack of focus in the implementation of these interventions [10] (ie, doctors do not see them as effective [11]), or it is due to the scarcity and inconclusive nature of the evidence published to date [12-14]. A recently published study offers recommendations on future interventions in this field, such as identifying the impact on the doctors’ workload [15].

The Catalan public health system consists of more than 160 providers that offer universal access to 7.5 million people, making it an integrated public welfare network that guarantees the universal right to health [16]. The large number of stakeholders has led centers to create their own information technology (IT) systems to meet specific needs. As a result, in 2008, the decision was made to implement a common platform that can securely share clinical information between different centers and health professionals [17]. Shortly afterward, the personal health folder (PHF), a tool that allows members of the public to securely access their personal information and online services [18,19], was deployed. eConsulta was subsequently launched in 2015 as an asynchronous tele-consultation tool for members of the public and general practitioners (GP) as a complement to face-to-face care. Its implementation has gradually extended to the entire network (more than 92% of primary care teams have used the tool). Nevertheless, its use in relation to conventional consultations remains low (accounting for just 0.9% of the total).

A recent study of factors that influence the use of eConsulta found that the main reason individuals used the service was to resolve administrative matters and because the service has potential for significantly reducing the number of face-to-face visits [20]. Another key factor in an effective analysis of the tool’s use is establishing the profile of the doctors who use it. Evidence suggests that specific characteristics determine the adoption of digital health technology. Studies have associated older age, close proximity to retirement, and female doctors with a lower probability of the GP using these tools [6,21,22]. Additionally, GPs with prior experience with other digital health technologies are shown to be more enthusiastic and optimistic than those who have not yet used them [23].

In light of this evidence, this study aimed to employ a multivariate logistic regression model to analyze the characteristics of GPs that affect their use of eConsulta in the context of the Catalan public health system.

## Methods

### Sample

**Figure 1:**
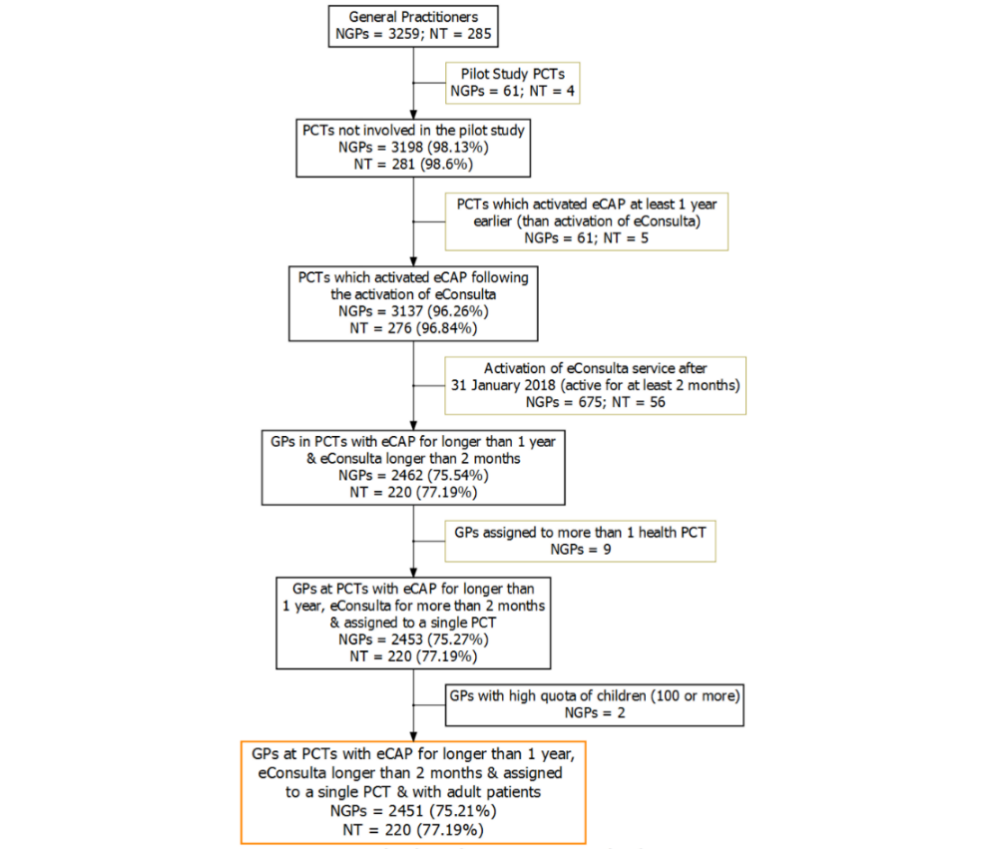
Flowchart of the study population. NGP: number of general practitioners; NT: number of primary health teams; PCT: primary health team; eCAP: primary care information system; GP: general practitioner.

This is a retrospective descriptive cross-sectional study of primary health care GPs belonging to the Catalan Health Institute (ICS), the major provider of primary care services in Catalonia (serving 74% of the Catalan population). The period of study was between January 1, 2016, and March 31, 2018. The target sample was made up of all 3259 GPs working at ICS from 285 centers. The following exclusion criteria were established: doctors belonging to centers participating in the pilot phase of the study, those belonging to centers that activated eConsulta less than 12 months after activating the electronic clinical IT system, GPs from centers that activated the eConsulta service after January 2018 (thus ensuring a minimum 2-month use of the service), those with more than 100 children assigned to them, and those who changed primary care teams during the study period. This study included a total of 2451 doctors serving 220 centers (Figure 1). Of these, 808 GPs who were excluded showed no statistically significant difference with respect to age, gender, and their quality of care (QoC) score, which is an indicator based on public information systems that evaluates performance related to the prevention and control of various illnesses such as hypertension, diabetes, and dyslipidemia (Table 1).

The main study variable was the use of the eConsulta service. Use was defined as any messages sent during the study period, and nonuse was defined as no messages sent. The following were considered independent variables: age, gender, socioeconomic level of the center, type of center (rural or urban), average number of adults attended, mean age of patients assigned to GP, percentage of patients who have activated their PHF, GPs involvement in teaching (yes or no), QoC score, pharmacy prescription quality standard (PPQS) score as of December 2017, and doctor’s accessibility (possibility of scheduling an appointment within 48 hours, 5 days, and 10 days).

**Table 1:** Sociodemographic characteristics of doctors included and excluded in the study.

Demographic			All (N=3259)		Excluded (n=808)	Included (n=2451)	*P* value
**Age, n (%)**							.16
		28-34 years	184 (5.65)		50 (6.19)	134 (5.47)	
		35-44 years	897 (27.50)		243 (30.10)	654 (26.70)	
		45-54 years	1001 (30.70)		227 (28.10)	774 (31.60)	
		55-66 years	1121 (34.40)		271 (33.5)	850 (34.70)	
		Missing	56 (1.72)		17 (2.10)	39 (1.59)	
**Sex, n (%)**							.61
	Male			2201 (67.50)	541 (67.00)	1660 (67.70)	
	Female			1002 (30.70)	250 (30.90)	752 (30.70)	
	Missing			56 (1.72)	17 (2.10)	39 (1.59)	
Quality of care score			760 (101)		756 (102)	762 (100)	.13

### Model

The descriptive analysis used the mean and standard deviation for continuous variables and numbers and percentages for categorical variables. The *t* test was used to test the significance for continuous variables, and the Chi-square test was used for categorical variables. To evaluate which variables make a doctor more likely to have used the platform, a multivariate logistic regression analysis was used with a significance level of 95%. R-3.5.1 (R Foundation for Statistical Computing, Vienna, Austria) software was used to conduct the analysis.

## Results

Table 2 shows the most prevalent characteristics of professional users. Doctors who use eConsulta have a higher percentage of patients who have activated their PHF and score higher on the PPQS score, QoC score, and accessibility of care indices. There are no statistically significant differences between doctors who use eConsulta and those who do not with respect to the average number of adults attended or the average age of the patients assigned to them.

The multivariate regression model examined which variables affect the use of eConsulta. These variables, independently related to the outcome, are distinct from those obtained from the bivariate analysis, in which they are combined. This means it was not possible to identify a specific correlation. The odds ratio for each outcome is shown with regard to the reference categories and can be interpreted as probabilities. A coefficient of less than 1 indicates that the use of eConsulta is less likely, while coefficients greater than 1 indicate a greater probability of the tool being used. Therefore, according to the regression model, the characteristics of the doctors that determine the use of eConsulta include the following: 45-54 years of age, a QoC score that is higher than the 80th percentile, a high degree of accessibility, are involved in teaching, and work in a primary care team in an urban area with a high socioeconomic level. All of the variables shown in Table 3 are statistically significant.

**Table 2:** Sociodemographic characteristics of doctors by use of eConsulta.

Demographic			Total (N=2451)		User (n=1269)	Nonuser (n=1182)		*P* value
**Sex, n (%)**							**<.001**	
	Female	1660 (67.70)		798 (62.90)		862 (72.90)		
	Male	752 (30.70)		439 (34.60)		313 (26.50)		
	Missing	39 (1.59)		32 (2.52)		7 (0.59)		
**Age, n (%)**							**<.001**	
	28-34 years	134 (5.47)		91 (7.17)		43 (3.64)		
	35-44 years	654 (26.70)		326 (25.70)		328 (27.70)		
	45-54 years	774 (31.60)		319 (25.10)		455 (38.50)		
	55-66 years	850 (34.70)		501 (39.50)		349 (29.50)		
	Missing	39 (1.59)		32 (2.52)		7 (0.59)		
**Type** **PCT^a^, n (%)**							**<.001**	
	0R (Rural)	227 (9.26)		145 (11.40)		82 (6.94)		
	1R (Semirural)	144 (5.88)		90 (7.09)		54 (4.57)		
	2R (Semiurban)	270 (11.00)		170 (13.40)		100 (8.46)		
	4U (Urban, very low socioeconomic level)	455 (18.60)		233 (18.40)		222 (18.80)		
	3U (Urban, low socioeconomic level)	474 (19.30)		246 (19.40)		228 (19.30)		
	2U (Urban, high socioeconomic level)	353 (14.40)		183 (14.40)		170 (14.40)		
	1U (Urban, very high socioeconomic level)	528 (21.50)		202 (15.90)		326 (27.60)		
**Type PCT, n (%)**							**<.001**	
	Rural	641 (26.20)		405 (31.90)		236 (20.00)		
	Urban	1810 (73.80)		864 (68.10)		946 (80.00)		
Adults seen, mean (SD)			1102 (229)		1111 (231)	1093 (226)		.06
Age quota, mean (SD)			50.1 (3.82)		49.9 (3.62)	50.3 (4.01)		.03
Quota for patients aged over 65 years (%), mean (SD)			23.6 (7.91)		23.2 (7.44)	24.1 (8.37)		.006
Patients with PHF^b^ activated (%), mean (SD)			5.49 (2.85)		4.62 (2.31)	6.41 (3.08)		<.001
**Teaching in 2017, n (%)**							**<.001**	
	No	2090 (85.30)		1123 (88.50)		967 (81.80)		
	Yes	361 (14.70)		146 (11.50)		215 (18.20)		
QoC^c^ score - December 2017, mean (SD)			762 (100)		749 (108)	775 (89.1)		<.001
**QoC score - December 2017 categorized, n (%)**							**<.001**	
	0-20	459 (18.70)		302 (23.80)		157 (13.30)		
	20-80	1497 (61.10)		734 (57.80)		763 (64.60)		
	80-100	495 (20.20)		233 (18.40)		262 (22.20)		
PPQS^d^ score - December 2017, mean (SD)			62.1 (18.4)		60.7 (18.6)	63.5 (18.1)		<.001
**PPQS score - December 2017 categorized, n (%)**							**<.001**	
	0-20	396 (16.20)		213 (16.80)		183 (15.50)		
	20-80	1364 (55.70)		672 (53.00)		692 (58.50)		
	80-100	477 (19.50)		203 (16.00)		274 (23.20)		
	Missing	214 (8.73)		181 (14.30)		33 (2.79)		
Replies in less than 5 days (%), mean (SD)			67.5 (28.60)		75.1 (29.80)	65.9 (28.10)		<.001
Accessibility in 48 hours (%), mean (SD)			31.1 (23.50)		31.7 (25.10)	30.5 (21.60)		.23
Accessibility in 5 days (%), mean (SD)			50.3 (27.80)		48.8 (28.80)	51.9 (26.60)		.006
Accessibility in 10 days (%), mean (SD)			74.2 (23.70)		71.5 (25.10)	77.1 (21.80)		<.001

^a^PCT: primary care team.

^b^PHF: personal health folder.

^c^QoC: quality of care.

^d^PPQS: pharmacy prescription quality standard.

**Table 3:** Results of the logistic regression model.

Demographic	Odds ratio (95% CI)	*P* values
Age: 35-44 years^a^	2.152 (1.431-3.277)	<.001
Age: 45-54 years	2.969 (1.979-4.512)	<.001
Age: 56-66 years	1.528 (1.019-2.320)	.04
Sex: Male	0.717 (0.592-0.869)	<.001
QoC^b^ score 20-80%	1.942 (1.542-2.454)	<.001
QoC score 80-100%	2.329 (1.761-3.088)	<.001
Semirural type	1.299 (0.823-2.047)	.26
Semiurban type	1.158 (0.784-1.713)	.46
Urban 4: very low socioeconomic level	2.024 (1.410-2.919)	<.001
Urban 3: low socioeconomic level	2.038 (1.428-2.920)	<.001
Urban 2: high socioeconomic level	2.207 (1.513-3.231)	<.001
Urban 1: very high socioeconomic level	4.016 (2.820-5.750)	<.001
Accessibility in 10 days	1.017 (1.013-1.021)	<.001
Teaching indicator 2017	1.496 (1.165-1.923)	.002

^a^All variables have a reference category.

^b^QoC: quality of care.

## Discussion

The results of this study differ from those of previous ones, which did not find significant differences in the gender and ages of doctors who adopted new technologies as part of their clinical practice [6,21,22]. In our sample, these differences can be partially attributed to characteristics of the Catalan ecosystem. For example, in Catalonia, GPs rarely obtain a stable position with their own patients before the former is 30 years of age. Likewise, the lower use of eConsulta in rural areas could be because, in Catalonia, patients’ access to health services in rural areas are better than in other regions due to the wide availability of local GP surgeons. The low level of use by younger doctors (30-44 years of age) could be explained by their relatively low level of confidence and security with respect to their patients, while the low level of use by older doctors (56-66 years of age) could be explained by their relatively lower levels of digital competency and their lower incentives to incorporate new elements into their practice due to the close proximity of retirement. The relationship between a higher use of the tool and higher QoC and PPQS scores could be attributed to the doctor’s confidence in adopting new tools. In relation to the higher use in urban areas (and possibly as a result of higher socioeconomic levels), it is worth mentioning that this study shows higher socioeconomic groups make more use of new technologies and have greater access to the internet. Primary care teams in areas with a high socioeconomic level have higher PHF activation rates than primary care teams in areas with lower socioeconomic levels

It seems that doctors who use eConsulta more have a higher level of accessibility for face-to-face visits. However, this might be because doctors who use eConsulta are probably more involved in managing their agenda and more prone to meeting QoC and PPQS. The increased waiting time for primary care in Catalonia warrants investigation in other studies.

Other policies may have acted as confounding factors that affected the interpretation of the results. For example, in January 2017, doctors in primary care teams in Barcelona were offered an economic incentive to use eConsulta. It should also be considered that in other instances, the Ministry of Health has introduced incentives to primary care teams throughout Catalonia to increase the use of the PHF.

In summary, these results show that being 45-54 years of age, having a QoC score higher than the 80th percentile, having a high degree of accessibility, being involved in teaching, and working in a primary care team in an urban area with a high socioeconomic level are characteristics that determine the use of tele-consultation in Catalonia. This study’s data cannot be extrapolated to other health systems; however, the results are critical for digital health policy planners, as the success of the tool will heavily depend on whether GPs promote it.

## Abbreviations

GP: general practitioners
ICT: information and communication technologies
IT: information technology
ICS: Catalan Health Institute
PHF: personal health folder
PPQS: pharmacy prescription quality standard
QoC: quality of care.

## References

[1] Banks J, Farr M, Salisbury C, Bernard E, Northstone K, Edwards H, Horwood J (2017). Use of an electronic consultation system in primary care: a qualitative interview study. Br J Gen Pract.

[2] Kierkegaard Patrick (2013). eHealth in Denmark: a case study. J Med Syst.

[3] Pearl R (2014). Kaiser Permanente Northern California: current experiences with internet, mobile, and video technologies. Health Aff (Millwood).

[4] Zhou YY, Kanter MH, Wang JJ, Garrido T (2010). Improved quality at Kaiser Permanente through e-mail between physicians and patients. Health Aff (Millwood).

[5] Anderson D, Villagra V, Coman EN, Zlateva I, Hutchinson A, Villagra J, Olayiwola JN (2018). A cost-effectiveness analysis of cardiology eConsults for Medicaid patients. Am J Manag Care.

[6] McGrail KM, Ahuja MA, Leaver CA (2017). Virtual Visits and Patient-Centered Care: Results of a Patient Survey and Observational Study. J Med Internet Res.

[7] Huygens MW, Vermeulen J, Friele RD, van Schayck OC, de Jong JD, de Witte LP (2015). Internet Services for Communicating With the General Practice: Barely Noticed and Used by Patients. Interact J Med Res.

[8] Brant H, Atherton H, Ziebland S, McKinstry B, Campbell JL, Salisbury C (2016). Using alternatives to face-to-face consultations: a survey of prevalence and attitudes in general practice. Br J Gen Pract.

[9] Hobbs FDR, Bankhead C, Mukhtar T, Stevens S, Perera-Salazar R, Holt T, Salisbury C (2016). Clinical workload in UK primary care: a retrospective analysis of 100 million consultations in England, 2007–14. The Lancet.

[10] Goldzweig CL, Orshansky G, Paige NM, Towfigh AA, Haggstrom DA, Miake-Lye I, Beroes JM, Shekelle PG (2013). Electronic patient portals: evidence on health outcomes, satisfaction, efficiency, and attitudes: a systematic review. Ann Intern Med.

[11] Farr M, Banks J, Edwards HB, Northstone K, Bernard E, Salisbury C, Horwood J (2018). Implementing online consultations in primary care: a mixed-method evaluation extending normalisation process theory through service co-production. BMJ Open.

[12] Atherton H, Pappas Y, Heneghan C, Murray E (2013). Experiences of using email for general practice consultations: a qualitative study. Br J Gen Pract.

[13] National Institute for Health and Care Excellence (2019). Evidence Standards Framework for Digital Health Technologies.

[14] WHO (2019). WHO Guideline: Recommendations on Digital Interventions for Health System Strengthening.

[15] Atherton H, Brant H, Ziebland S, Bikker A, Campbell J, Gibson A, McKinstry B, Porqueddu T, Salisbury C (2018). The potential of alternatives to face-to-face consultation in general practice, and the impact on different patient groups: a mixed-methods case study. Health Serv Deliv Res.

[16] García Altés A (2017). Desigualdades Socioeconómicas en Salud.

[17] Fernández O, Domínguez CO, Alés XB (2017). Acceso de los pacientes a su historia clínica electrónica: ventajas e inconvenientes para pacientes y profesionales. FMC - Formación Médica Continuada en Atención Primaria.

[18] La Meva Salut (Personal Health Folder).

[19] Departament de Salut (2014). Model D'Atenció No Presencial en el Sistema Sanitari de Catalunya.

[20] López Seguí F, Vidal-Alaball J, Sagarra Castro M, Garcia Altés A, Garcia Cuyàs F (2020). Does teleconsultation reduce face to face visits? Evidence from the Catalan public primary care system. JMIR Preprints.

[21] Lupiañez Villanueva F, Folkvord F, Fauli C (2018). Benchmarking deployment of eHealth among general practitioners. RAND.org.

[22] Li Junhua, Talaei-Khoei Amir, Seale Holly, Ray Pradeep, Macintyre C Raina (2013). Health Care Provider Adoption of eHealth: Systematic Literature Review. Interact J Med Res.

[23] Antoun J (2016). Electronic mail communication between physicians and patients: a review of challenges and opportunities. Fam Pract.

